# Clinical features of visual disturbances secondary to isolated sphenoid sinus inflammatory diseases

**DOI:** 10.1186/s12886-017-0634-9

**Published:** 2017-12-06

**Authors:** Lanlan Chen, Libin Jiang, Bentao Yang, Prem S. Subramanian

**Affiliations:** 1 Department of Ophthalmology, Hainan Branch of Chinese People’s Liberation Army General Hospital, Sanya, Hainan China; 2 Beijing Ophthalmology and Visual Sciences Key Laboratory, Beijing Tongren Eye Center, Beijing Tongren Hospital, Capital Medical University, Beijing, China; 3 Department of Radiology, Beijing Tongren Hospital, Capital Medical University, Beijing, China; 4 Department of Ophthalmology, University of Colorado School of Medicine, Aurora, CO USA; 5 Department of Ophthalmology, Beijing Tongren Hospital, Capital Medical University, No.1 Dongjiaominxiang Street, Dongcheng District, Beijing City, 100730 China

**Keywords:** Visual disturbance, Diplopia, Headache, Isolated sphenoid sinus inflammatory diseases

## Abstract

**Background:**

Visual disturbances associated with isolated sphenoid sinus inflammatory diseases (ISSIDs) are easily misdiagnosed due to the nonspecific symptoms and undetectable anatomical location. The main objective of this retrospective case series is to investigate the clinical features of visual disturbances secondary to ISSIDs.

**Methods:**

Clinical data of 23 patients with unilateral or bilateral visual disturbances secondary to ISSIDs from 2004 to 2014 with new symptoms were collected. Collected data including symptoms, signs, neuroimaging and pathologic diagnosis were analyzed.

**Results:**

There were 14 males and 9 females, and their ages ranged from 31 to 83 years. Fifteen patients suffered blurred vision and 11 patients suffered binocular double vision, including 3 patients who had unilateral visual changes and diplopia simultaneously. Headache was observed in 18 patients, and orbit pain/ocular pain in 8 patients. Other presenting symptoms included ptosis (4 patients) and proptosis (1 patient). Only 5 patients had nasal complaints. The corrected visual acuities were between NLP to 20/20. Patients with diplopia included 5 with unilateral oculomotor nerve palsy and 6 with unilateral abducens nerve palsy. All patients performed orbital/sinus/brain radiologic examination and found responsible lesions in sphenoid sinus. All patients underwent endoscopic sinus surgery, and 9 patients were found to suffer sphenoid mucocele, 9 with fungal sinusitis, and 5 with sphenoid sinusitis. Visual disturbances improved in 6 patients, and all the patients with diplopia had a postoperative recovery.

**Conclusion:**

Visual disturbances resulting from ISSIDs are relatively uncommon, but it is crucial that the patient with new vision loss or diplopia and persistent headache or orbit pain be evaluated for the possibility of ISSIDs especially before corticosteroid administration.

## Background

Isolated sphenoid sinus inflammatory diseases (ISSIDs) describe a collection of several pathologic entities, including sphenoid sinusitis (in which the majority is acute and bacterial sinusitis), sphenoid sinus mucocele, and chronic/ acute fungal infection. The sphenoid sinus lies in close proximity to important anatomic structures, [1] including the cavernous sinus, the optic nerve, the internal carotid artery, and cranial nerves III, IV, V, and VI. Thus, spread of infection or inflammation beyond the sphenoid sinus to these neighboring structures may result in serious intracranial and orbital complications and potentially irreversible or fatal neurological sequelae. The mortality is as high as 40%-80% in acute fungal sinusitis patients with orbit or intracranial invasion [2, 3]. However, the early symptoms of sphenoid sinus diseases are often nonspecific, and routine examinations offer little diagnostic information on sphenoid pathology, resulting in inappropriately delayed diagnosis and treatment. About 24%-50% patients with ISSIDs may present with decreased vision [4] and failure to consider sphenoid sinus disease as a possible cause may lead to delayed diagnosis. It should be noted that sphenoid disease in the presence of other sinus disease can also cause visual symptoms and needs to be addressed just as urgently, but it’s an another entity and will not be discussed here. Ophthalmologists thus have a very important role in detecting and diagnosing ISSIDs. Therefore, we retrospectively investigated the clinical features of the visual disturbances secondary to ISSIDs.

## Methods

A retrospective electronic medical records review was performed on all patients who visited initially to the Ophthalmology Department and then were treated by the Otolaryngology Department of Beijing Tongren Hospital, from January, 2004 to November, 2014. Approval from Institutional Review Board and Ethics Committee of Capital Medical University for retrospective studies was obtained before commencing the study. The study followed the tenets of the Declaration of Helsinki. Only patients who had presenting symptoms of decreased vision and/or double vision and diagnosed as isolated sphenoid sinus disease were selected. Generally, the patients had CT/ MRI of orbit/ sinus/ head examinations performed when the ophthalmologists tried to discover the cause of diplopia and/or optic neuropathy which was thought to be responsible for decreased vision based on the clinical picture. When sphenoid sinus lesions were noted on radiographic imaging, patients were referred to Otolaryngology Department because visual disturbances were thought to be related to the sphenoid sinus lesions. Each patient underwent endoscopic surgery as a principal treatment, and postoperative management included antimicrobial treatment, regular debridement and endoscopic examination. All specimens suggested isolated disease of sphenoid sinus. The diagnosis of isolated sphenoid sinus disease was made by otolaryngologists based on characteristic signs and symptoms, routine ear, nose, and throat examinations, radiographic imaging (CT or MRI), and histopathological examinations of the resected specimens. Exclusionary criteria were patients who had sinusitis of other paranasal sinuses or pathology arising from other sinuses and spreading into the sphenoid sinus, patients with malignant tumors, and patients had other ophthalmic or systemic disorders that were the cause of their visual symptoms. Demographic data, clinical symptoms, interval between the onset of visual disturbances and operation, physical signs, imaging studies (CT/ MRI of orbit/ sinus/ head), surgical findings, pathological reports and initial ophthalmic diagnosis were analyzed. Patients with incomplete or unavailable medical records were excluded.

## Results

**Fig. 1:**
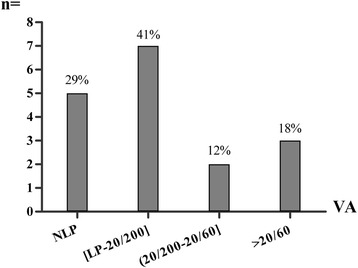
Decreased vision secondary to isolated sphenoid sinus inflammatory lesions. VA, Visual Acuity; NLP, No Light Perception; LP, Light Perception

**Fig. 2:**
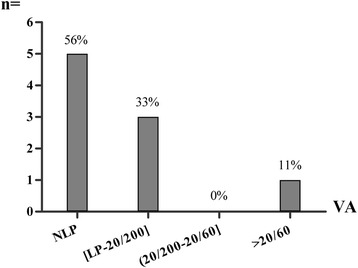
Decreased vision secondary to fungal sphenoid sinusitis. VA, Visual Acuity; NLP, No Light Perception; LP, Light Perception

Among 67 patients who presented with symptoms of decreased vision and/or double vision and were diagnosed with isolated sphenoid sinus disease, 23 subjects were included in the present report (Table 1). Fourteen patients (61%) were male and 9 (39%) were female. Age ranged from 31 to 83 years old (mean 54.9 ± 15.8 years), with 10 patients (43%) older than 60 years of age. The interval, initial ophthalmic diagnosis, accompanying symptoms, radiologic findings, pathological diagnosis, and outcomes are summarized in Table 2. Table 3 shows the clinical manifestations in patients with fungal sphenoid sinusitis. The severity of visual loss secondary to isolated sphenoid sinus inflammatory lesions and fungal sphenoid sinusitis are shown respectively in Fig. 1 and Fig. 2.

**Table 1:** Clinical information in patients with visual disturbances secondary to ISSIDs

No.	Age/Sex	Complaint	Signs (best corrected visual acuity/other ocular signs)	Initial diagnosis	Radiologic finding	Pathological finding	outcome
1	47/M	gradually blurred vision of right eye for 3 mo	0.1/RAPD(+), light optic disc of right eye	optic neuropathy (R)	CT: bony destruction, MRI: heterogenous(T1), hyperintense(T2), (SS)	SS	No improvement
2	65/F	blurred vision of right eye for 1 mo	0.01/dilated pupil, RAPD(+)	optic neuritis (R)	CT: bony erosion, MRI: isointensity(T1), slight hyperintensity(T2), (SS)	FSS	No improvement
3	56/F	gradually blurred vision of right eye for 3 mo	NLP/RAPD(+), pallor of the right optic disc	optic neuritis (R)	CT: bony destruction, MRI: isointensity(T1), hypointensity(T2), (FSS)	FSS	No improvement
4	73/F	blurred vision of left eye for 3 mo	HM/30 cm/ RAPD(+), light optic disc of left eye	optic neuropathy (L)	CT: bony pressure erosion, MRI: hypointensity (T1), hyperintense(T2), (SSM)	SSM	No improvement
5	51/M	gradually blurred vision of right eye for 1 y	0.3/ RAPD(+), light optic disc of right eye	optic neuropathy (R)	CT: bony pressure erosion, MRI: hypointensity (T1), hyperintense(T2), (SSM)	SSM	improvement
6	62/M	blurred vision of right eye for 4 y, accompanied with ipsilateral protopsis	0.5/proptosis, RAPD(+), pale optic disc of right eye	optic nerve atrophy (R)	CT: bony pressure erosion, MRI: hypointensity (T1), hyperintense(T2), (SSM)	SSM	No improvement
7	61/F	sequential blurred vision of both eye for 1 mo, blind for 1 w	NLP,NLP/mydriasis, pallor of the both optic disc	optic neuropathy (B)	CT: bony destruction with calcification, MRI: isointensity(T1), hypointensity(T2), (FSS)	FSS	No improvement
8	79/M	sudden blurred vision of right eye for 6 d	NLP/ RAPD(+)	optic neuritis (R)	Brain CT: normal, MRI: isointensity(T1), hyperintense(T2), (SS)	FSS	Partially improvement
9	47/M	blurred vision of left eye for 3 d	0.3/−	optic neuritis (L)	CT: low-density soft tissue mass, MRI: hypointensity (T1), hyperintense(T2), (SSM)	SSM	Improvement
10	39/F	gradually blurred vision of left eye for 2 mo, blind for 1 w	NLP/RAPD(+), pallor of the left optic disc	optic neuropathy (L)	CT: low-density mass, bony sclerosis, (FSS)	FSS	No improvement
11	52/F	gradually blurred vision of right eye for 1 y	0.6/ light optic disc of right eye	optic neuropathy (R)	CT: low-density mass, bony sclerosis, (FSS)	FSS	Improvement
12	33/M	blurred vision of both eye, progressive impairment of right eye for 5 d	0.05,1.0/ right RAPD(+)	optic neuritis (B)	CT: low-density soft tissue mass, MRI: isointensity(T1), hyperintensity (T2), (SSM)	SSM	Improvement
13	74/F	blurred vision of left eye and binocular double vision for 20 d	0.1 /ptosis, dilated pupil, exotropia and restricted adduction of left eye	optic neuropathy (L); incomplete CN III palsy(L); orbital apex syndrome(L)	CT: bony sclerosis, CT/MRI: normal, (SS)	FSS	Both improvement
14	45/M	binocular double vision for 2 mo, blurred vision of left eye for 3 d	0.02/ left RAPD(+), esotropia and restricted abduction of left eye	optic neuropathy(L); incomplete CN VI palsy(L)	CT: low-density soft tissue mass, bony sclerosis, MRI: hyperintensity(T1), hyperintensity(T2), (SS)	SS	Improvement of eye movement
15	83/M	progressively blurred vision of right eye for 1 mo, and used to be binocular double vision for a time	0.05/ ptosis, restricted adduction, mydriasis, pale optic disc of right eye	optic neuropathy(R); incomplete CN III palsy(R)	CT: soft tissue mass, bony sclerosis and thickening, (SS)	FSS	Improvement of eye movement and ptosis
16	33/M	binocular double vision for 1 mo	restricted adduction of right eye	incomplete CN III palsy(R)	CT: bony destruction, MRI: hypointensity(T1), hyperintense(T2), (SSM)	SSM	Improvement
17	31/M	sudden binocular double vision for 20 d	restricted abduction of right eye	incomplete CN VI palsy(R)	CT: bony pressure erosion, with focal destruction, MRI: isointensity(T1), slight hyperintensity(T2), (SS)	FSS	Improvement
18	67/M	sudden binocular double vision for 1 mo	restricted abduction of left eye	incomplete CN VI palsy(L)	CT: bony sclerosis, (SS)	SS	Improvement
19	32/F	sudden binocular double vision for 7 d	ptosis and restricted adduction of left eye	incomplete CN III palsy(L)	CT: bony destruction, MRI: isointensity(T1), hyperintensity(T2), (SS)	SS	Improvement
20	46/M	sudden binocular double vision for 1 mo	restricted abduction of right eye	incomplete CN VI palsy(R)	MRI: hypointensity (T1), hyperintense(T2), (SSM)	SSM	Improvement
21	62/M	binocular double vision for 3 y	ptosis and restricted abduction of right eye	incomplete CN III palsy(R)	CT: low-density soft tissue mass, MRI: hypointensity(T1), hyperintense(T2), (SSM)	SSM	Improvement
22	51/F	sudden binocular double vision for 28 d	the left eye fail to abduct	CN VI palsy(L)	CT: low-density soft tissue mass without bony changes, (SS)	SS	Improvement
23	74/M	sudden binocular double vision for 16 d	the right eye fail to abduct	CN VI palsy(R)	CT: low-density soft tissue mass, MRI: hypointensity(T1), hyperintense(T2), (SSM)	SSM	Improvement

Note: The pathological diagnosis of the only one patient (case 6) suffered exophthalmos in this study was sphenoid sinus mucocele and the exophthalmos had improved after treatment, although the visual acuity had no improvement

*M* male, *F* female, *R* right, *L* left, *B* bilateral, *SS* sphenoid sinusitis, *FSS* fungal sphenoid sinusitis, *SSM* sphenoid sinus mucocele

**Table 2:** Clinical manifestations in patients with visual disturbances secondary to ISSIDs

Visual disturbance	No. of patients	Interval^a^		Initial Ophthalmic diagnosis	Accompanying symptoms			Radiologic findings			Pathologic diagnosis			Outcome (improvement)		
		≤1 month	>1 month		Headache (followed/accompanied by ocular symptoms)	ocular pain	nasal symptoms	FSS	SSM	SS	FSS	SSM	SS	FSS	SSM	SS
unilateral visual disturbance	15 (17 eyes) ^b^	8	7	Optic neuropathy (9)	13 (7/6)	6	3	4	5	6	8	5	2	3 (3 eyes)	3 (4 eyes)	0
				Optic neuritis (5)												
				Optic nerve atrophy (1)												
bilateral visual disturbance	11 ^b^	9	2	incomplete CNIII palsy (5)	8 (4/4)	3	2	0	4	7	3	4	4	3	4	4
				Incomplete CNVI palsy (4)												
				complete CNVI palsy (2)												

Note: ^a^The interval between the onset of visual disturbance and operation

^b^Among them, there were three patients had visual loss and diplopia simultaneously

*SS* sphenoid sinusitis, *FSS* fungal sphenoid sinusitis, *SSM* sphenoid sinus mucocele

**Table 3:** Clinical manifestations in patients with fungal sphenoid sinusitis

No.	Age/Sex	Visual Loss (VA)	Diplopia	Accompanying Symptoms		Underlying Diseases	Interval^b^	Outcomes	
				Pain^a^/Localization	Nasal Symptom			Visual Loss	Diplopia
2	65/F	R(2/200)	–	+/temporal, occipital	–	–	1 mo	No improvement	/
3^c^	56/F	R(NLP)	–	+/temporal,peri-orbital	–	rheumatoid arthritis	3 mo	No improvement	/
7^c^	61/F	R(NLP) L(NLP)	–	+/vertex,occipital	–	DM(U)	1 mo	No improvement	/
8	79/M	R(NLP)	–	+/frontal,retro-orbital	–	DM(U), ischemic heart disease	6 d	improvement	/
10	39/F	L(NLP)	–	+/temporal	–	DM(U)	2 y	No improvement	/
11	52/F	R(20/30)	–	+/occipital	nasal obstruction	–	1 y	improvement	/
13	74/F	L(20/200)	+	+/vertex	–	IGT, hypothyroidism	20 d	improvement	improvement
15	83/M	R(10/200)	+	+/occipital, retro-orbital	–	–	1 mo	No improvement	improvement
17^c^	31/M	–	+	+/vertex,occipital	bloody rhinorrhea	carriers of chronic hepatitis B virus	20 d	/	improvement

Note: ^a^All patients had persistent ipsilateral headache

^b^The interval between the onset of visual disturbance and operation

^c^Case 3 had been worked in a leather factory (warm and humid conditions) for 2 months (12 working hours/d), and had a history of high-dose steroids therapy because of rheumatoid arthritis. Case 7 refused to administrate anti-fungal agents postoperatively. Case 17 had been worked in a construction site year in year out, in which the diet and housing conditions were poor

*R* right eye, *L* left eye, *M* male, *F* female, *VA* visual acuity, *NLP* no light perception, *DM* diabetes mellitus, *U* uncontrolled, *IGT* impaired glucose tolerance

The symptomatology was often nonspecific. The ocular symptoms occurred in the form of unilateral visual disturbance (blurred vision, 15/23), bilateral visual disturbance (binocular diplopia, 11/23), ptosis (4/23) and proptosis (1/23). Pain was the most frequent symptom, presenting in 20 (87%) of the 23 patients, including headache (18 patients) and ocular pain (8 patients). Specifically, there were 14 patients who suffered persistent and refractory headache since the onset of symptoms. Only 5 patients presented with nasal symptoms (including rhinorrhea, nasal obstruction and bloody rhinorrhea).

On initial examination, the population consisted of eight patients with unilateral optic neuropathy, one patient with bilateral optic neuropathy, four patients with unilateral optic neuritis, one patient with bilateral optic neuritis, one patient with unilateral optic nerve atrophy, five patients with unilateral incomplete oculomotor nerve palsy, four patients with unilateral incomplete abducens nerve palsy, and two with unilateral complete abducens nerve palsy. Among them, there were three patients had unilateral optic neuropathy and diplopia simultaneously.

**Fig. 3:**
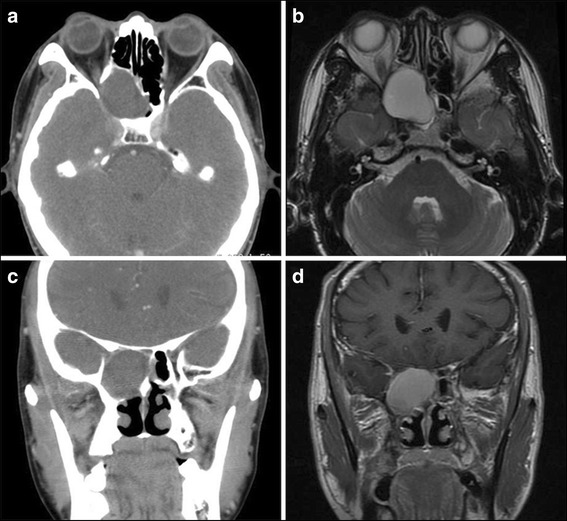
Case 5, male, 51y, sphenoid sinus mucocele (right, VA = 30/200). Neuroimaging showed a hemispherical abnormal mass rose from right sphenoid sinus with expansion to surrounding areas. There is extension into the right orbital apex and compression of the right optic nerve. **a**: Axial CT scan showing the expansile lesion. **b**: Axial, T2-weighted MRI scans with homogenous, fluid density lesion in the right sphenoid sinus. **c**: Coronal CT scan shows chronic bony expansion of the sinus. **d**: Coronal, T1-weighted, contrast-enhanced MRI scan demonstrating the fluid-filled lesion

**Fig. 4:**
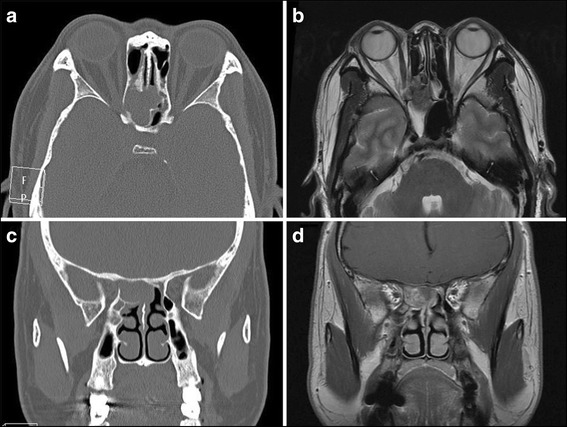
Neuroimaging for case 1, male, 47y, chronic sphenoid sinusitis (right, VA = 20/200). **a**: Axial computed tomography scan shows bony destruction at the right orbital apex. **b**: Axial, T2-weighted magnetic resonance imaging scan reveals a homogenous, hyperintense lesion of the inferior aspect of the right sphenoid sinus. **c**: Coronal computed tomography scan demonstrating erosion of the bony medial orbital apex. **d**: Coronal, T1-weighted, contrast-enhanced magnetic resonance imaging scan without contrast shows heterogenous signal in the right sphenoid sinus

**Fig. 5:**
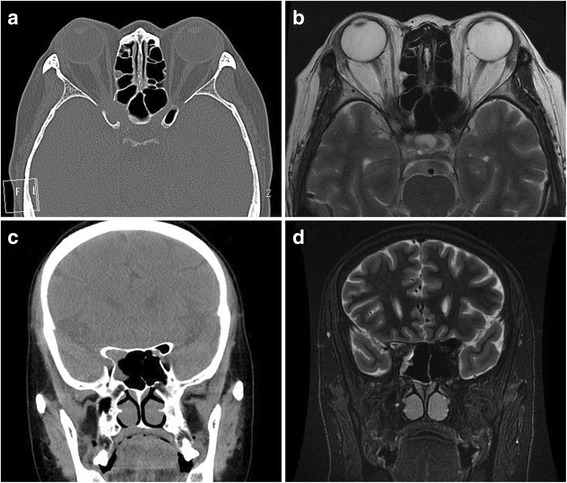
Neuroimaging for case 3, female, 56y, invasive fungal sphenoid sinusitis (right, VA = no light perception). **a**: Axial CT scan with expansion and opacification of the right optic canal and optic nerve, which was invaded by fungal organisms. **b**: Axial, T2-weighted MRI scan showing hypointense signal primarily adjacent to the right optic canal. **c**: Coronal CT scan showing opacification of the right anterior clinoid process. **d**: Coronal, T2-weighted, contrast-enhanced MRI scan with abnormal signal in the area of the right orbital apex

Twenty-two subjects underwent CT/MRI scans of the orbits/sinuses/brain; imaging was interpreted as indicating fungal sinusitis in 4 cases, sphenoid sinus mucocele in 9 cases, and bacterial or inflammatory sphenoid sinusitis in 10 cases (Figs. 3, 4 and 5). All patients were treated by endoscopic sinus surgery to remove or drain the sphenoid sinus lesion, and the postoperative pathology was reviewed and compared with the preoperative radiologic diagnosis. The correct initial diagnosis was made in 18/23 (78.3%) cases (chronic fungal sphenoid sinusitis in 4 cases- case 10 and case 11, acute fungal sinusitis in 2 cases- case 3 and case 7, sphenoid sinusitis in 5 cases, mucocele in 9 cases), and the remaining 5 subjects had fungal sinusitis, all of whom were diagnosed preoperatively with nonspecific sphenoid sinusitis (cases 2, 13, 15 and 17 were finally diagnosed as chronic fungal sinusitis, and case 8 was diagnosed as acute fungal sinusitis).

All patients received additional medical treatments after surgery including antifungal drugs where indicated. Upon discharge, vision had improved in seven of the seventeen eyes (41.2%) with decreased visual acuity, including 3 eyes with fungal sphenoid sinusitis; 2 of these 3 had a short duration of vision loss (6 and 20 days), and the third had good initial visual acuity (VA = 20/30) despite 1-year disease duration preoperatively (case 11). The remaining 4 eyes (case 5, 9, 12), with vision loss from mucocele, regained vision as well. Three of the 4 eyes (cases 9, 12) had a short duration of 3 or 5 days (cases 9 and 12), while case 5 had only moderately (20/60) preoperative visual loss. All patients with diplopia had improvement but the degree of recovery was variable.

## Discussion

Optic neuropathy from ISSIDs may arise from spread of sinus inflammation and infection, compression by an expansible lesion, or ischemia [5]. Visual loss may be acute or subacute, and the fundus often is normal or shows mild optic disc swelling, which might result in a misdiagnosis of “optic neuritis”. In the present series, patients who suffered vision loss without ptosis were all diagnosed as optic neuritis or optic neuropathy initially. The manifestations of optic neuropathy secondary to ISSIDs differ from the optic neuritis associated with multiple sclerosis (MS), also called typical optic neuritis (ON). Typical ON most commonly affects women less than 50 years old, and usually presents with subacute painful loss of vision in one eye. The pain usually localizes in or around one eye and commonly worsens with eye movements. The severity of the pain varies, but it is unusual for it to disturb sleep or last longer than a few days. Spontaneous recovery of vision begins within three to 5 weeks after onset, and systemic high-dose corticosteroid administration can hasten the recovery of visual acuity [6, 7]. In our study, all 15 patients with optic neuropathy from ISSIDs have no gender differences, in which the ratio of men and women almost equally divided. Patients suffered persistent headache, migraine, or pain around the eye. The visual acuity decreased progressively, showing no response to steroid therapy or only brief improvement. These characteristics fit atypical ON, which differs greatly from typical ON. For this entity, it is crucial to perform brain or orbital radiologic imaging and serum laboratory tests, especially before the use of systemic corticosteroids or immunosuppressive agents [8, 9]. In diagnosis of ISSIDs-associated optic neuropathy, the significance of the radiologic workup is far more important than serum laboratory tests. Adequate brain and/or orbital imaging were critical in making the diagnosis of ISSIDs in this series. It is notable that different pathogens of ISSIDs may have similar features on brain or orbital MRI. Five patients in our series interpreted as sphenoid sinusitis through brain or orbital MRI turned out to be fungal infection pathologically. It is commonly difficult to distinguish between fungal infection of sphenoid sinus and other sphenoid inflammation according to MRI features alone, but the former always causes bony changes [4, 10], thinning or thickening, which may be shown on CT. Mucoceles can cause bony loss via pressure/expansion. Therefore, sinus CT should be performed if brain or orbital MRI shows sphenoid sinusitis. If adjacent bony destruction is found on CT, infection by fungus or tumor formation should be highly suspected, and biopsy should be performed as soon as possible.

Sphenoidotomy with drainage is the main procedure to diagnose and treat the ISSIDs [11]. Treatment with steroids alone before clearing the infectious lesions may facilitate the spread of infection, especially in acute fungal infection, and may even result in life-threatening intracranial spread; therefore, steroid should only be used once the underlying infectious lesion has been excluded or treated. A small dose of steroid may be used postoperatively to reduce swelling if needed. In the present study, only 7/17 eyes experienced slight visual acuity improvement postoperatively, similar to previous reports [12, 13]. The poor prognosis of visual acuity in ISSIDs may result from the severe injury of optic nerve and the coexistence of multiple injury mechanisms including direct nerve infiltration by inflammation and infection, compression, and ischemia. Despite the limited efficacy of visual function in long duration cases of sinusitis by surgery, it remains important in preserving the retained visual function, improving or relieving headache, and preventing the further spread of infection or inflammation.

The binocular diplopia from ISSIDs arises with involvement of one or more cranial nerve(s) and/or extraocular muscles by infiltration or compression from the inflammatory lesion. In the 11 cases of binocular visual disturbance in the present study, 5 patients presented with incomplete unilateral oculomotor nerve palsy, 4 with incomplete unilateral abducens nerve palsy and 2 with complete unilateral abducens nerve palsy. Two of the five patients with incomplete unilateral oculomotor nerve palsy also had ipsilateral optic nerve injury due to the involvement of orbital apex and presented with incomplete orbital apex syndrome. In the other three subjects, injury of oculomotor nerve in the region of orbit, direct injury of extraocular muscles, or both may have led to double vision. For patients with abducens nerve palsy, we surmise that inflammation from the sphenoid sinus spread to the abducens nerve in cavernous sinus, where the abducens nerve runs close to the sphenoid sinus [14]. All the patients with diplopia in the present study had remarkable improvement of double vision after debridement of sinus lesions. Furthermore, the improvement in diplopia was much better than the improvement in visual acuity of optic nerve injury. It is possible that the ocular motor nerves are more likely to recover from this type of injury than is the optic nerve, and subjects with diplopia also may undergo neuroimaging earlier in their disease course than those patients with vision loss alone.

When presents as orbital apex syndrome, some important entities should be differentiated. Cavernous sinus thrombosis (CST) is a life-threatening disorder that can complicate facial infection, sinusitis, orbital cellulitis, especially in the setting of a thrombophilic disorder. Early signs of CST include fever, headache, and ophthalmic involvements, which are nearly universal, including periorbital edema, lid erythema, ptosis, proptosis, restricted or painful eye movement, and less commonly papilledema, decreased visual acuity [15]. High-resolution contrast-enhanced head CT typically shows cavernous sinus expansion and irregular filling defects as direct signs [16]. Tolosa-Hunt syndrome is another differential disease and is described as unilateral orbital pain associated with paresis of one or more of the III, IV and/or VI cranial nerves, which resolves spontaneously but may recur. Episodic orbital pain often localized around the ipsilateral brow and eye. Clinically, Paresis coincides with the onset of pain or follows it within 2 weeks and resolve within 72 h when treated adequately with corticosteroids [17]. Granulomatous inflammation of the cavernous sinus, superior orbital fissure or orbit, demonstrated by MRI or biopsy are important in differential diagnosis [18].

There are some limitations in this study. Firstly, there was not complete information about the exact visual acuity outcomes since the patients were discharged from the Department of ENT and did not have a later ophthalmologic examination. Prospective enrollment of patients with ISSIDs may allow us to identify factors that influence final visual outcome in the short and long-term, and we propose to evaluate such factors in future research.

## Conclusion

In conclusion, although visual disturbances resulting from ISSIDs are relatively uncommon, it is important to understand the clinical features of visual disturbances secondary to ISSIDs to avoid the misdiagnosis and mistreatment. Whenever patients with manifestations of atypical optic neuritis, particularly those with severe or persistent headache and/or orbit pain, are evaluated, neuroimaging to include the orbits, sinuses, and brain must be performed expeditiously to allow identification of the underlying causes, especially before administration of systemic corticosteroids.

## Abbreviations

ISSIDs: Isolated sphenoid sinus inflammatory diseases
MS: Multiple sclerosis
ON: Optic neuritis
